# What is the protective effect of preischemic kisspeptin-10 administration against ischemia/reperfusion injury of striatum on mice?

**DOI:** 10.55730/1300-0144.5493

**Published:** 2022-08-04

**Authors:** Hatice AKKAYA, Engin SÜMER, Selim KUTLU, Hatice SOLAK, Bayram YILMAZ

**Affiliations:** 1 Department of Biochemistry, Faculty of Pharmacy, University of Health Sciences, İstanbul, Turkey; 2 Experimental Research Center, Faculty of Medicine, Yeditepe University, İstanbul, Turkey; 3 Department of Physiology, Faculty of Medicine, Necmettin Erbakan University, Konya, Turkey; 4 Department of Physiology, Faculty of Medicine, Yeditepe University, İstanbul, Turkey

**Keywords:** Cerebral ischemia/reperfusion injury, kisspeptin, neurotransmitter, mouse, oxidative stress

## Abstract

**Background/aim:**

Kisspeptin is a neuropeptide with a primary role on the onset of puberty and has beneficial effects on ischemia/reperfusion (I/R) injury. In this study, we aimed to investigate the effect of kisspeptin administration on striatal I/R injury in mice.

**Material and methods:**

Forty adult C57/BL6 mice were randomly divided into four groups: Sham, Kisspeptin, I/R, and I/R + Kisspeptin groups. The groups were administered with either physiological saline (Sham and I/R groups) or kisspeptin (Kisspeptin and I/R + Kisspeptin groups) intraperitoneally 40 min before the operation. A microdialysis probe was placed in the right striatum according to stereotaxic coordinates. During the experimental period, artificial cerebrospinal fluid was passed through the micropump. Then, transient cerebral ischemia was established by compressing both common carotid arteries with an aneurysm clip for 15 min and animals were reperfused for 2 h. Throughout the process of microdialysis (before, during and after I/R period), samples were collected to measure dopamine (DA), noradrenaline (NA), and 3,4-dihydroxyphenylglycine (DHPG) at intervals of 20 min continuously. At the end of the reperfusion period, the animals were decapitated, striatum was dissected, half of the animals were used for oxidative stress analyses (reduced glutathione, glutathione S-transferase (GST), superoxide dismutase (SOD), malondialdehyde (MDA), and the other half were used for histopathology analyses.

**Results:**

Number of glial cells was significantly increased in kisspeptin-administered groups. DA levels in ischemic animals were decreased by kisspeptin administration (p < 0.0001). NA levels were reduced in animals administered with kisspeptin without I/R injury (p < 0.05). DHPG levels reduced during the reperfusion period in ischemic animals (p < 0.05). Kisspeptin did not exhibit a significant antioxidant activity in the ischemic animals, while GST and SOD levels were reduced in the I/R + kisspeptin group compared to the kisspeptin group (p < 0.05).

**Conclusion:**

Our results suggest that kisspeptin may be regulating the neurotransmitter release and metabolism, as well as inflammatory response in brain upon I/R injury.

## 1. Introduction

Cerebral ischemia/reperfusion (I/R) injury is a known complication of returning blood flow to ischemic brain tissue, and secretion of proteases and free radicals to the wounded area is a result of the reintroduction of oxygen and white blood cells via blood flow [1]. Cerebral I/R injury frequently occurs in patients with cerebral palsy upon reintroduction of blood flow, leads to severe neurological deficits [2]. Pharmacological investigations have indicated by brain microdialysis studies that neurotransmitters may be released upon ischemia [3,4].

The prefrontal cortex circuits travel along a portion of the basal ganglion-caudate nucleus, globus pallidus, and thalamus [5]. One of the main nuclei forming the basal ganglia is the striatum that consists of caudate nucleus, putamen, ventral striatum and the striatum entering the basal nucleus [5]. The dorsolateral prefrontal cortex [6], orbitofrontal cortex [7], and dorsal anterior cingulate cortex have projections to the striatum that play a role in behavioural flexibility [8], where disruptions can lead to various behavioural or emotional deficits [9,11]. Impaired catecholamine activity may also lead to circuitry dysregulation in these regions [12,14].

Kisspeptin receptor (Kiss1r) is found in various regions in the brain including pons, midbrain, thalamus, hypothalamus, hippocampus, amygdala, cortex, frontal cortex, and striatum as well as in the peripheral organs such as liver and intestine [15]. Kisspeptin, which is mainly released from the hypothalamus [15], is also secreted from the cerebral cortex and hippocampus [16,17]. In addition, it was reported that peripheral kisspeptin can reach GnRH neurons and pass through the blood brain barrier [18]. Kisspeptin plays a role in vasoconstriction besides its neurological functions. In the rat leg ischemia model, kisspeptin improved blood flow, induced capillary growth, and suppressed cell growth in vivo and in vitro detected with laser doppler. [19]. Several kisspeptin isoforms including kisspeptin-10, -13, and -54 were detected in the human coronary artery and umbilical vein [20]. All cleavage products of kisspeptin activate Kiss1R signalling, but kisspeptin-10 (Kisspeptin) has been shown to be the most physiologically active peptide [21,22].

It is known that the striatum is more vulnerable than the cortex in ischemia [23,24], and kisspeptin may exert a protective effect on the striatum during ischemia by acting on catecholamines. Therefore, in this study, the effects of kisspeptin on levels of dopaminergic and noradrenergic neurotransmitters before, during, and after transient cerebral ischemia were determined by brain microdialysis. In addition, oxidative stress markers and histological alterations were investigated.

## 2. Materials and methods

### 2.1. Animals and experimental setup

Experiments were performed in accordance with National Health Institute of Health Guidelines for the Care and Use of Laboratory Animals and ARRIVE guidelines with local government approval. Ethical approval was obtained from Local Ethics Committee on Experimental Animal Research (Approval number: 700). Forty young adult male C57/BL6 mice weighing 20–25 g were used in this study. As the menstrual cycle of the female animals and hormonal changes may have significant impact on the brain chemistry, male animals were used in the study. Animals were housed under controlled temperatures (21 ± 1°C) and controlled lighting conditions (12-h light/dark cycle). Standard mouse chow and tap water were provided ad libitum.

**Figure 1:**
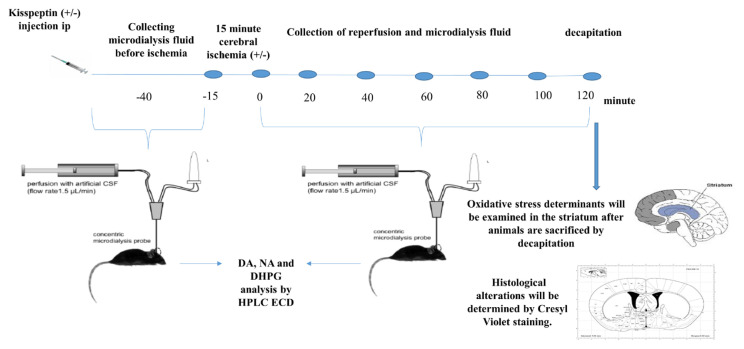
Experimental design. The mice were placed on the stereotaxic frame under anaesthesia, the microdialysis probe was placed in the right striatum according the coordinates determined by the Mouse Brain in Stereotaxic Coordinates by Paxinos and Franklin [25] (AP: 0.02 mm, ML: 2 mm, DV: 3 mm). Artificial cerebrospinal fluid was circulated through the micropump during the experimental period. The flow rate of artificial cerebrospinal fluid was set at 1.5 uL. Three microlitres of HCl was added to the tubes that were claimed at the beginning of the microdialysis procedure for degradation of monoamines by monoamine oxidase inhibition. Microdialysis fluids were collected twice at 20-min intervals as indicated by blue circles.

The mice were anaesthetized with isoflurane (Piramal, USA) and placed on the stereotaxic frame, the microdialysis probe was placed in the right striatum according to the coordinates determined by the Mouse Brain in Stereotaxic Coordinates (antero-posterior = 0.02 mm, medial-lateral = 2 mm, dorsal-ventral = 3 mm) by Paxinos and Franklin. (Figure 1) [25]. Experimental groups were as follows: Sham (n = 10), Kisspeptin (n = 10), I/R (n = 10), I/R + Kisspeptin (n = 10). I/R groups (I/R and I/R + Kisspeptin groups) received either 200 uL physiological saline (Sal) or 20 nmol kisspeptin intraperitoneally [26] (M2816 Metastin 45–54 amide, human, SIGMA, France) and the artificial cerebrospinal fluid (aCSF) was prepared as indicated previously [27] and passed through the micropump (CMA, Sweden) during the experimental period. The flow rate of aCSF was set at 1.5 μL. Three microlitres of HCl was added to the tubes at the beginning of the microdialysis procedure to avoid the degradation of monoamines by monoamine oxidase inhibition. Microdialysis fluids were collected twice at 20-min intervals. At the end of this period, an aneurysm clip was used to squeeze both of carotid arteries for 15 min (Figure 1) and one microdialysis fluid was obtained and then 2 h of reperfusion was performed [28, 29], during which six microdialysis fluid samples were collected. In total, 6 μL of sample is collected in the tubes. A total of nine samples were obtained from each mouse (two samples before ischemia, one sample during ischemia, and six samples after reperfusion). In the other two groups (Sham and Kisspeptin), the same operations were performed except for the I/R procedure. Study was completed within 6 months (31.09.2018–31.03.2019).

After collecting microdialysis fluid samples at nine time points, the samples were stored at −80 °C for dopamine (DA), noradrenaline (NA) and 3,4-dihydroxyphenylglycol (DHPG) analyses. After decapitation, the striatum was dissected from the brain tissue. Reduced glutathione (GSH), glutathione S-transferase (GST), malondialdehyde (MDA), and superoxide dismutase (SOD) levels were measured to determine oxidative stress in the striatum. In addition, the brain tissues of the other five animals were used for the determination of histological alteration in the striatum by cresyl violet staining.

### 2.2. Histological study

At the end of the experimental period, the mice were decapitated and the brain tissues were frozen on dry ice. Tissues were sectioned coronally at 20 μm thickness using a Leica CM1850 UV Cryostat and immersed in different alcohol concentrations (100%, 95%, and 70%, respectively) for 5 min. Samples were stained with cresyl violet (0.5 g/mL, Sigma, France) for 5 min. After the staining, the slides were treated with different concentrations of alcohol (70%, 95%, and 100%, respectively) for 5 min and xylene for 20 min. Afterwards, sections were observed under a microscope with a fixed digital camera (Zeiss, Germany) at 40× magnifications. The total number of cells was counted in different groups at 150× magnifications. Images of sections were examined, and cell counting was performed by Fiji ImageJ software 1.5.2i (USA). Large cells with pale nuclear staining are marked as neurons and smaller round or oval nuclei without stained cytoplasm are considered glia. Small, elongated nuclei are considered epithelial cells and not included in our counts [30]. Glial fibrillary acidic protein (GFAP) staining was not performed since all glial cells would be evaluated rather than just the distribution of astrocytes [31].

### 2.3. Catecholamine analyses

Analytical high pressure liquid chromatography column (HPLC, Agilent 1260 USA) with an electrochemical detector (Waters 2465, USA) system was used for catecholamine analysis. The temperature of the column furnace was fixed at 40 °C. Flow rate at the HPLC was set at 1 mL/min during analyses. Injections of 20 μL were performed. The modified catecholamine mobile phase solution with 32 mM citric acid (Sigma-Aldrich, USA), 16 mM sodium citrate (Sigma-Aldrich, USA), 0.9 mM sodium 1-heptanesulfonic acid (Sigma-Aldrich, USA), 0.16 mM EDTA (Sigma-Aldrich, USA), 36 mM tetrahydrofuran (VNR), 0.02 mM glacial acetic acid (Merc, Germany), and 0.616 mM Methanol (Sigma-Aldrich, USA) at pH 4.9 [32] were continuously ran into the system.

### 2.4. Biochemical analyses

GSH, GST, MDA, and SOD measurements were made spectrophotometrically. Changes in the levels of MDA in the striatum were analysed as reported by Placer et al. [33]. The GSH and GST determinations were conducted as reported by Elman et al. [34] and Habig et al. [35], respectively. SOD determinations were conducted according to the method by Sun et al. [36]. Protein concentrations were determined using the Lowry method [37].

### 2.5. Statistical analyses

The data were expressed as mean ± standard deviation (SD). Statistical analyses were conducted by using GraphPad^®^ Prism statistical analysis software (GraphPad Software, Inc., USA, ver. 7.0). Number of glia and neuronal cells were analysed by using one-way analysis of variance (ANOVA) followed by Tukey’s multiple comparison test. Catecholamine levels were analysed by using two-way ANOVA followed by Tukey’s multiple comparison test. GSH, GST, MDA, and SOD levels were analysed by using one-way ANOVA followed by Tukey’s multiple comparison test. A p-value lower than 0.05 was accepted as statistically different.

## 3. Results

### 3.1. Histological Assessment of the effect of Kisspeptin on I/R injury in striatum by cresyl violet staining

**Figure 2:**
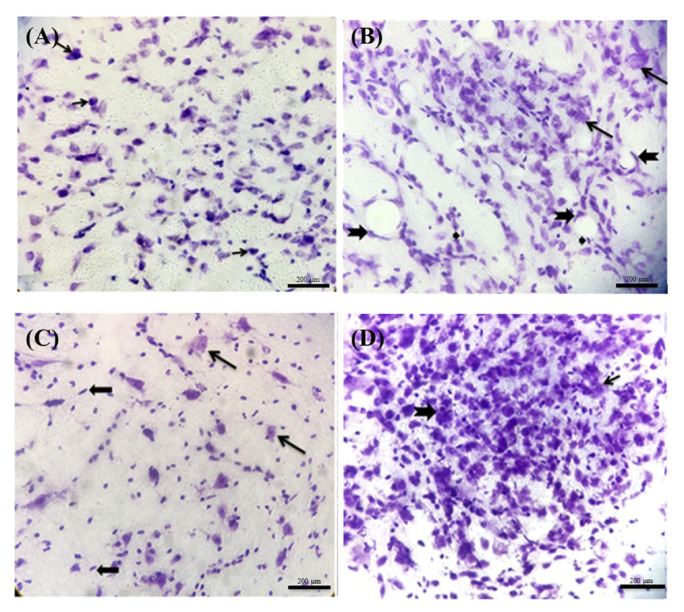
Histological assessment of the effect of kisspeptin on the ischemic injury in striatum by cresyl violet staining**. (a)** In the Sham group, neuronal pyknosis were observed as darkly stained (thin arrows). **(b)** In the Kisspeptin group, eosinophilic shrunken cells (thin arrows), microglial cells (arrowheads), and endothelial cells (thick arrows) were observed. **(c)** In I/R group, shrunken neuronal cell bodies were observed and they became eosinophilic. Damaged tissue with minimal microglial cells (thick black arrows) were detected, as well as marked neuronal loss and gliosis in the striatum. **(d)** I/R + Kisspeptin group diffused activation of microglial nodule (thick arrows) and reduced pyknotic cells were detected (thin arrows). Scale bars of the images obtained at 40× magnification represent 200 μm in length.

**Figure 3:**
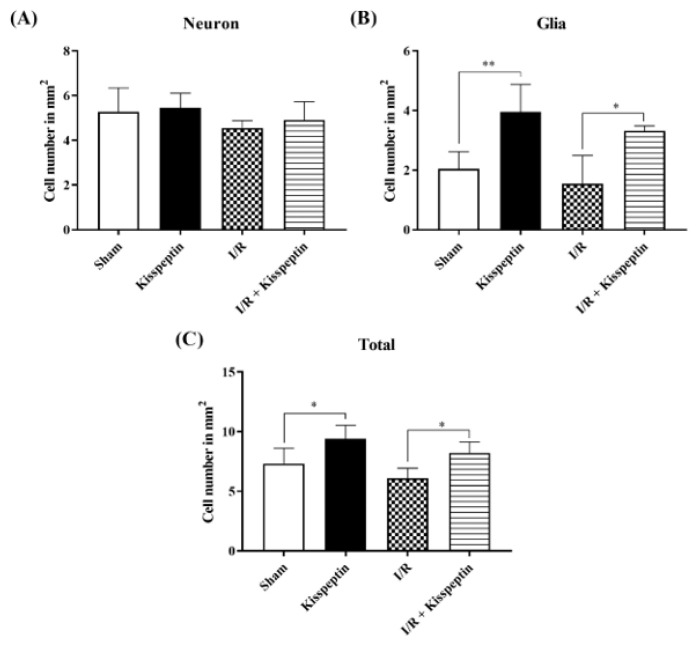
Assessment of the effect of kisspeptin on the ischemic injury in striatum on cell number. **(a)** Neuron number, **(b)** glia number, and **(c)** total cell number. Statistical analyses: One-way ANOVA followed by Tukey’s multiple comparison test (*p < 0.05 and **p < 0.01).

Histological assessments of the ischemic injury in the corpus striatum revealed that I/R increased gliosis, while small amount of pyknosis was also observed in the Sham group (Figures 2A and 2C). Microglial and endothelial cells were observed in Kisspeptin group (Figure 2B). On the other hand, reduced amounts of pyknotic cells were observed in the I/R + Kisspeptin group, as well as reduced ischemic damage (Figure 2D). The cell counting results revealed that the number of the neurons did not change significantly in any of the group at the end of experimental period (Figure 3A; p > 0.05). A small but significant increase in the glial cells was observed in Kisspeptin and I/R + Kisspeptin groups (Figure 3B; p < 0.01 and p < 0.05, respectively). The total cell numbers were also found to be increased in both Kisspeptin and I/R + Kisspeptin groups (Figure 3C; p < 0.05).

### 3.2. Effect of transient cerebral ischemia and Kisspeptin administration on catecholamine levels

**Figure 4:**
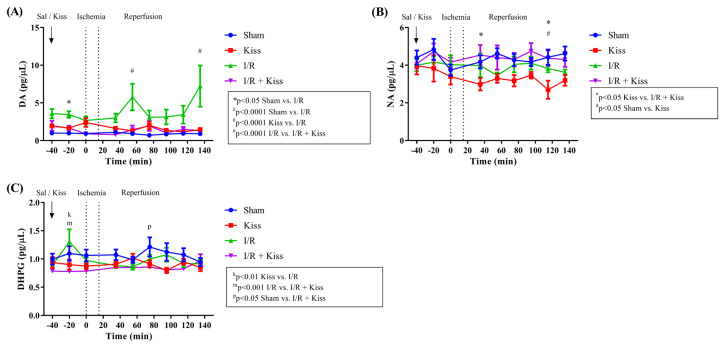
Effect of kisspeptin administration on catecholamine levels. **(a)** DA, **(b)** NA and **(c)** DHPG levels were measured from microdialysis samples. Data are mean ± SD (n = 10 in each group). Statistical analyses: Two-way ANOVA followed by Tukey’s multiple comparison test.

During the preischemic period, significantly high DA levels were found in the I/R group compared to the Sham group (*p_(t = -20)_ < 0.05) likely due to the individual differences in the animals in both groups (Figure 4A). No significant differences were observed from the other groups during the preischemia period (Figure 4A). During the reperfusion period, DA levels were significantly higher in the I/R group than the other groups (^#^p_(t = 55 and 135)_ < 0.0001). DA levels were found similar in kisspeptin administered I/R animals (I/R + Kisspeptin) with the DA levels in the Sham and Kisspeptin groups (Figure 4A).

With regards to NA levels, there were no significant differences between the groups during the preischemia period (Figure 4B). The Kisspeptin group exhibited significantly lower NA levels during the reperfusion period (at t = 35 min and t = 115 min) compared to the I/R + Kisspeptin group (*p < 0.05) and the Sham group (^#^p_(t=115)_ < 0.05; Figure 4B).

During the preischemic period at t = −20, the Ischemia group exhibited significantly higher DHPG levels than the Kisspeptin group (p < 0.01) and I/R + Kisspeptin group (p < 0.001; Figure 4C). DHPG levels of the Sham group were significantly higher than those of the I/R + Kisspeptin group at t = 75 min (^p^p < 0.05; Figure 4C).

### 3.3. GSH, GST, MDA, and SOD levels

The effects of carotid artery ischemia on the oxidative stress parameters in the striatum were determined (Table 1). GSH levels were not affected, while nearly significantly lower GSH levels were determined in the Kisspeptin group when compared to the Sham group (Table 1; p = 0.055). The I/R + Kisspeptin group exhibited significantly lower GST levels compared to the Kisspeptin group (Table 1; p < 0.05). MDA levels were not significantly altered when compared to each other (Table 1; p < 0.01). SOD levels were significantly lower in the I/R + Kisspeptin group than the Kisspeptin group. No significant differences in the oxidative stress parameters were observed between the I/R and I/R + Kisspeptin groups.

## 4. Discussion

In this study, we observed that kisspeptin administered before transient cerebral ischemia increased glia and total cell number after I/R. Changes in metabolic activity suggest that kisspeptin may play a role in I/R damage by regulating neurotransmitter release, metabolism, and inflammatory activity in the infarct area.

Ischemic damage causes a variety of neuronal cell death [38,39]. Eosinophilic necrosis of the neuron [40,41], gliosis, and neuronal death were previously presented in different ischemia models [42,43]. Additionally, induced inflammation upon I/R is also observed [44,45]. Previous studies suggested that kisspeptin is a protector against neuronal damage caused by oxidative stress [26]. Similarly, kisspeptin acts protective against oxidative stress in spermatogenic cells [46,47] and liver tissue [48]. Although GST and SOD levels were altered in I/R and I/R + Kisspeptin group of animals, we did not observe any significant improvement in antioxidant activity altered by I/R injury.

Glial cells are the key immune cells of the brain [49] upon ischemic injury, they become activated and can be detected in the injury area as early as 2 h [50]. In addition, microglia are activated and migrate to the damaged area upon ischemic stroke [49]. At the early stages of ischemia, microglial cells are in M1 polarization status, and M1 polarized microglia release proinflammatory mediators associated with tissue damage, while those in the M2 polarization status release antiinflammatory mediators associated with tissue healing [49]. In this context, kisspeptin may have a role in the polarization of the microglial cells towards M2 status, leading to neuroprotection and tissue repair by only slightly altering the number of glial cells. However, the role of kisspeptin on the inflammatory processes needs to be elucidated. In addition, no glial polarization markers were investigated in our study; therefore, it would be relevant to investigate the polarization profiles of glial cells by kisspeptin in I/R injury.

As known, the neuropeptide kisspeptin encoded by *Kiss1* regulates reproduction by stimulating GnRH secretion. Kiss1-expressing neurons are found primarily in the hypothalamic anteroventral periventricular (AVPV / PeN) and arcuate nucleus of hypothalamus (ARC) [51]. In mice, tyrosine hydroxylase (TH), the rate-limiting enzyme in the catecholamine synthesis, is coexpressed in most of the AVPV/PeN *Kiss1* cells [52]. Although the intense colocalization of *TH* and *Kiss1* in AVPV/PeN has been illustrated, the function of DA produced in these cells was not clearly identified, and it was suggested that it might not be essential for puberty or reproduction processes [52]. However, a previous study showed that DA plays an inhibitory role on the GnRH secretion by suppressing the LH pulses regulated by kisspeptin neurons in ARC [53]. Administration of GnRH analogue, alarelin, was shown to reduce the apoptotic cell death in the CA1 region of the hippocampus [54]. On the other hand, it was reported that peptides such as arginine vasopressin (AVP) and gonadotropin inhibitory hormone (GnIH) and neurotransmitters such as glutamate and γ-aminobutyric acid (GABA) regulate AVPV kisspeptin neurons, and AVPV kisspeptin neurons express galanin and dopamine together [55].

Kisspeptin was previously indicated to have beneficial effects upon I/R in the uterus and ovary [56]. Another study suggested that there is a correlation between the decreased cardiac kisspeptin levels and ischemic heart disease [57]. On the other hand, kisspeptin was suggested to suppress endothelial cell growth likely via induction of cellular senescence [19]. As the DA release and reuptake is impaired in I/R injury [58], kisspeptin may be regulating the DA reuptake by glial cells [59] as Kiss1r is expressed in glial cells [60]; however, the knowledge regarding the role of Kiss1r in glial cells is very limited.

Various psychosocial and physical stresses activate peripheral sympatho-adrenomedular and central catecholaminergic systems to increase circulating epinephrine and norepinephrine secretion. These stress hormones, known as catecholamines, trigger a series of biological reactions such as increased energy metabolism to prepare the organism to cope with stressful events and adapt [61]. Changes in metabolic activity represent the severity and type of the brain ischemia and determine the amount of neurotransmitter release upon ischemia. Excessive neurotransmitter release is strongly associated with the neuronal damage and death upon cerebral ischemic damage [62,63]. It has been shown that NMDA receptors are overstimulated due to excessive glutamate release [64,65], and other monoamine neurotransmitters such as DA, serotonin (5-HT) [66,67] and NA [68,69] are released upon cerebral ischemia and play roles in ischemic damage. A previous study in gerbils showed that the regional turnover of homovanillic acid (HA)/DA increased 15 min after 1 h of bilateral brain ischemia [70]. In another study in which middle cerebral artery occlusion was performed, the protective effect of administration of the β-adrenoceptor antagonist, esmolol, and landiolol reduced the infarct area in rats although the glutamate levels were reduced, increased NA levels remained unchanged [71]. It was indicated that glutamate stimulates the NA release in different regions of the brain [72,73]. NA depletion was reported to improve brain recovery from ischemia-induced damage in rats [74,75], and additionally, NA was found to modulate the Kiss1 transcript, Kiss protein levels, by acting on α-1 adrenergic receptors [76].

In our study, we observed decreased NA levels in Kisspeptin group but not in I/R + Kisspeptin group. Previously, an increase in the metabolism of NA into DHPG [77, 78] in the neocortex, CA3, and dentate gyrus were observed in rats 1 h after ischemia [79]. Similarly, a significant increase in DHPG levels was found in the forebrain of rats after ischemia, suggesting a higher NA turnover rate upon ischemia [80]. In addition, upon myocardial ischemia, an increase in NA then in DHPG levels during the ischemic period and a decrease in NA levels during the reperfusion period were observed [81]. Interestingly, in our study, the I/R group had significantly higher DHPG levels at the 20th min of the preischemic period (t = −20) than the other groups. No differences were observed in animals until 1 h after reperfusion. Additionally, sham animals exhibited significantly higher DHPG levels at t = 75 compared to the Kisspeptin and I/R + Kisspeptin groups, while DHPG levels remained unchanged at the end of the experimental period. While these results suggest that kisspeptin may regulate both NA release and metabolism upon ischemia, these mechanisms need to be clarified with a longer reperfusion period.

As a subgroup of glial cell populations, astrocytes have important roles in ischemic injury [82] by regulating homeostatic balance [83], supplying tropic factors for neuronal protection [84] and alleviating oxidative stress [85]. Additionally, GnRH activity and secretion are regulated by glia [86]. Glia-derived factors such as prostaglandin E_2_ [87] and transforming growth factor-β [88] influence the activity of GnRH neurons. On the other hand, ischemia is known to increase NA levels but NA levels decrease during reperfusion [89]. Several studies have shown that glial activation is mediated by NA [90], while astrocytes uptake NA [91,92]. Additionally, astrocytes and synaptic structures are located in close distance, leading to the exposure of astrocyte to neurotransmitters released from the synaptic terminals [93]. That may be due to the fact that glial cells uptake the synaptic NA and represents it to the other glial cells via noradrenaline-specific receptor. In our study, after ischemia, the reduction in NA and subsequent increase in glial population levels in the kisspeptin group could be explained by that kisspeptin might regulate the NA uptake and NA-specific receptor signalling, leading to microglial recruitment. On the other hand, it is required to elucidate which specific glial populations are affected by kisspeptin after I/R injury.

In conclusion, our results suggest that kisspeptin may play a role in I/R injury by regulating neurotransmitter release, metabolism, and inflammatory activity in the infarct area. However, one should be cautious in interpretation when describing the beneficial effects of Kisspeptin on I/R. In addition to mechanical studies, longer reperfusion times are needed to observe the long-term effects of Kisspeptin on I/R injury.

## Figures and Tables

**Table 1:** Effect of ischemia and kisspeptin on GSH, GST, MDA, and SOD levels. Data are mean ± SD (n = 10 in each group except for the Sham group in MDA analysis, n = 9). Statistical analyses: One-way ANOVA followed by Tukey’s multiple comparison test. Data sharing the same superscript letters are significantly different than each other.

	Sham	Kisspeptin	I/R	I/R + Kisspeptin	p-value (ANOVA)
GSH (umol/mL)	14.34 ± 5.79	7.86 ± 4.69	11.27 ± 3.44	12.06 ± 7.40	0.083
GST (μmol/min.mg protein)	13.73 ± 5.54	15.67 ± 7.83 a	11.97 ± 4.04	7.74 ± 2.47 a	0.014
MDA (nmol/g)	1.74 ± 0.88	1.83 ± 0.95	1.64 ± 0.83	2.66 ± 1.14	0.085
SOD (U/mg protein)	0.44 ± 0.20	0.61 ± 0.27 b	0.42 ± 0.17	0.26 ± 0.08 b	0.003

a p < 0.05; Kisspeptin group vs I/R + kisspeptin group

b p < 0.01; Kisspeptin group vs I/R + kisspeptin group
